# Decreased Functional Connectivity Between the Left Amygdala and Frontal Regions Interferes With Reading, Emotional, and Executive Functions in Children With Reading Difficulties

**DOI:** 10.3389/fnhum.2020.00104

**Published:** 2020-04-28

**Authors:** Ohad Nachshon, Rola Farah, Tzipi Horowitz-Kraus

**Affiliations:** ^1^Educational Neuroimaging Center, Faculty of Education in Science and Technology, Faculty of Biomedical Engineering, Technion – Israel Institute of Technology, Haifa, Israel; ^2^Reading and Literacy Discovery Center, Cincinnati Children’s Hospital Medical Center, Cincinnati, OH, United States

**Keywords:** anxiety, emotional state, executive functions, functional connectivity, reading, reading task, reading difficulties, resting state

## Abstract

**Introduction:**

Dyslexia is a reading disorder characterized by significant difficulty in reading, as well as reports of altered executive functions (EF). Children with reading difficulties (RD) experience a broad range of social and emotional problems. Recently it was suggested that children with RD have altered functional connections within the amygdala, which is related to emotional processing. Altered brain laterality related to reading was previously reported in children with RD. Hence, we sought to determine the differences in functional connectivity between the right and left emotional network as related to emotional challenges and the other reported difficulties in reading and EF in children with RD compared to typical readers.

**Methods:**

Sixty-four 8 to 12 year old children, 27 children with RD and 37 age-matched typical readers, participated in the study. Reading, emotional, and EF abilities were assessed. Global efficiency of the emotional network was calculated and compared between the groups, and left vs. right functional connectivity of the amygdala was tested using the CONN toolbox. Functional connectivity measures were then associated with measures of reading, emotional, and EF abilities.

**Results:**

Children with RD showed significantly decreased emotional and EF abilities compared to typical readers. A negative correlation between reading, emotional, and EF abilities was determined in both groups. Neuroimaging results showed decreased global efficiency measures within the emotional network in children with RD, who also showed lower functional connectivity between the amygdala and the left and right frontal pole regions. Results also indicated increased functional connectivity of the right vs. left amygdala with left and right pre-central and post-central gyri regions, which were related to decreased reading, emotional, and EF abilities in both typical readers and children with RD.

**Conclusion:**

The positive relationship between EF and emotional abilities in children with RD strengthens the relationship between EF difficulties and emotional stress, which in turn may lower EF abilities (monitoring, inhibition, and attention) as well as decreased reading abilities. The emotional challenges in children with RD were associated with decreased functional connectivity of the left amygdala with pre/post central gyrus and cognitive-control regions. These findings suggest that although the right hemisphere is thought to be related to emotional stress, it was the decreased control of the left hemisphere that was related to emotional disturbance in children with RD.

## Highlights

– Decreased emotional and cognitive control abilities and the relationship between these abilities in children with RD vs. typical readers.
– Decreased global efficiency of the emotional network during rest in children with RD vs. typical readers, related to lower emotional abilities.
– Increased functional connectivity between the left vs. right amygdala and the right frontal pole, and between the left amygdala and the left frontal pole in typical readers compared to children with RD.
– Increased functional connections between the left vs. right amygdala in typical readers compared to children with RD, related to better cognitive control, and emotional and reading abilities.

## Introduction

### Emotional Difficulties in Children With Reading Difficulties

Dyslexia is a reading disorder characterized by significant difficulty in reading that is not explained by any other intelligence, motivational, or environmental deficit (International Dyslexia Association [IDA], 2011). Recent studies suggest additional alterations in cognitive control, also called executive functions (EF), may indicate a common malfunction in EF in individuals with reading difficulties (RD) (Habib, 2000), with some suggesting the EF challenge as the source for the reading-based difficulties shared by children with RD (Pennington, 2006). Interestingly, it was also found that children with RD experience emotional problems including low self-esteem, anxiety, and depression (Grills-Taquechel et al., 2012). We recently demonstrated that in addition to their impaired reading, children with RD demonstrated decreased emotional and EF abilities compared to typical readers, which were also positively correlated with each other (Nachshon and Horowitz-Kraus, 2018). Cleary, children with RD face several challenges and it is challenging to objectively identify each one. With the development of imaging technologies, a better understanding of the neural circuits involved in these RD challenges is emerging. Therefore, the goal of the current research study was to determine the neurobiological correlates for the emotional difficulties reported in children with RD.

### Neural Circuitry Involved in Reading Difficulties

Evidence of the neurobiological, neuropsychological, and neurophysiological basis for RD has existed since the end of the 19th century (Habib, 2000). Traditional studies suggested that a decreased activation in “classical” reading-related neural circuits, such as the left fusiform gyrus, is related to orthographical processing (see, for example, Pugh et al., 2000). Children with RD showed greater activation in frontal lobes, i.e., in regions responsible for EF, during a narrative comprehension task compared to typical readers (Horowitz-kraus et al., 2016). Decreased functional connectivity between reading and EF regions (i.e., anterior cingulate cortex) during reading in children with RD compared to typical readers was also observed (Horowitz-Kraus and Holland, 2015). Interestingly, several studies have indicated a right-lateralization during reading in these children, specifically in reading-related regions such as the right fusiform gyrus, suggesting a heavier reliance on the right hemisphere for reading as opposed to the left fusiform gyrus in children with RD compared to typical readers (Horowitz-Kraus et al., 2014). Recent studies also reported differences in functional connectivity related to cognitive control networks in children with RD. Levinson et al. reported greater functional connectivity in children with RD between the EF network and visual, language, and cognitive control regions during the Stroop task (Levinson et al., 2018). They suggested that children with RD might exploit neural circuits supporting EF when performing a cognitive task more than typical readers. Nevertheless, since emotional and cognitive abilities were found to be linked (Ochsner and Gross, 2005), a question arises as to the neurobiological signatures for the emotional difficulties in children with RD. This study set out to find these signatures, if they exist.

### Neural Circuitry Related to Emotional Difficulties

Emotional difficulties such as anxiety, stress, and depression are currently defined using the Diagnostic and Statistical Manual of Mental Disorders (American Psychiatric Association [APA], 2013). Anxiety is characterized by continuous excitation due to expectations of bad outcomes or constant threat (Kim et al., 2011). Previous neuroimaging studies reported that increased amygdala activation was correlated with higher anxiety levels. For example, Bishop et al. (2004) reported that increased amygdala activity when faced with unattended fearful faces was correlated with higher levels of self-reported anxiety. Etkin et al. (2004) revealed a positive association between the unconscious processing of fearful faces by the basolateral amygdala and subjects’ trait anxiety levels. Finally, anxiety was associated with elevated amygdala activity when faced with threat-related stimuli and non-threat-related stimuli, suggesting that elevated amygdala activity may reflect greater anxiety levels even in the absence of a clear threat (Somerville et al., 2004).

Recent work indicated the contribution of subregions of the amygdala to emotional and reading abilities in children with RD. Increased functional connectivity between the amygdala and the medial prefrontal cortex (mPFC) in children with RD compared to typical readers was demonstrated (Davis et al., 2018). Children with RD exhibited a positive functional connectivity between the left basolateral amygdala and the mPFC compared to a negative functional connectivity between these regions in typical readers. Children with RD and typical readers showed similar functional connectivity between the bilateral centro-medial amygdala and the mPFC. This connectivity was negatively correlated with reading ability, whereas functional connectivity between the right centro-medial amygdala and the left mPFC were positively correlated with anxiety symptoms in the entire sample. The authors theorized that a similar pattern of unequal functional connectivity exists in individuals with anxiety disorder and this reinforces the co-occurrence of emotional and reading difficulties in children with RD. The authors, however, did not examine the relationship between the amygdala laterality, i.e., right vs. left functional connections with other regions in the brain related to reading or cognitive control, as related to reading or emotional abilities in children with RD. Due to the overall role of the right hemisphere in processing emotional information (Borod et al., 1988), and based on previously observed laterality in children with RD, specifically as related to reading words (i.e., decreased activation of the left occipital regions in children with RD during reading) (Horowitz-Kraus and Breznitz, 2014), an explicit examination of the amygdala’s laterality (i.e., the relationship between the right vs. left amygdala functional connections with other regions in the brain), and reading, emotional, and EF abilities in children with RD is warranted. Such an examination may elucidate the overall different lateralization in children with RD related to their reading, emotional, and EF abilities.

The overarching goal of this study was to close the gap in knowledge regarding the neurobiological evidence for the involvement of emotional difficulties in children with RD, specifically focusing on the functional connections between the left vs. right amygdala and the entire brain related to reading and emotional abilities. We hypothesized that children with RD would show decreased functional connectivity patterns within the emotional network compared to typical readers during rest. We also anticipated that children with RD would show different functional connectivity in the left vs. right amygdala compared to typical readers, which would be related to decreased reading, emotional, and EF abilities in children with RD.

## Materials and Methods

### Participants

Sixty-four 8 to12 year old children [27 children with RD (mean age = 10.16, SD = 1.11, 15 females) and 37 age-matched typical readers (mean age = 9.93, SD = 1.05, 15 females)] participated in the study (no significant age difference between the group, *t* = 0.829, ns). All participants were in the normal range of non-verbal IQ tests, as measured by the test of non-verbal intelligence (TONI) (Brown et al., 1997). No differences were found in general verbal ability, as tested by the Peabody picture vocabulary test (PPVT) (Dunn and Dunn, 2007), between children with RD and typical readers.

All participants were native English speakers, Caucasian, with average socioeconomic status. All were right-handed, displayed normal or corrected-to normal vision in both eyes and had normal hearing. All children were without a history of neurological or emotional disorders, and no differences were found between the two reading groups in attention ability [as measured using the Conners questionnaire (Conners, 1989)]. Participants were recruited from posted online ads and through commercial advertisements. All participants signed informed written assent and their parents provided informed written consent prior to inclusion in the study; all were compensated for their participation. The Cincinnati Children’s Hospital Medical Center (CCHMC) Institutional Review Board approved the study. All participants underwent thorough baseline behavioral (reading, EF) and emotional (anxiety questionnaire) testing. The data were collected at the Pediatric Imaging Research Consortium (PNRC) at CCHMC in Cincinnati, Ohio. Behavioral data acquisition (reading, EF, and emotional questionnaires) lasted approximately 2 h.

### Reading Measures

Children with RD were assigned to the RD group based on a previous diagnosis. Their reading ability was verified using a set of reading tests (Nachshon and Horowitz-Kraus, 2018), on which they had to score in less than the 25th percentile for at least two of the subtests. Children assigned to the typical readers group were age-matched students without previous reports of impaired reading and who scored in greater than the 25th percentile for all of the reading tests performed.

To evaluate their reading ability, the children’s reading was tested using the following: Word level reading (1) Automatic word reading accuracy/orthography, the Test of Words Reading Efficiency [TOWRE (Torgesen et al., 1999)]; (2) Automatic decoding, the pseudowords reading efficiency subtest from the TOWRE; (3) Non-timed word reading accuracy/orthography (Letter–Word subtest) (Woodcock et al., 1989); and (4) Non-timed decoding of pseudoword reading (Word–Attack subtest) (Woodcock et al., 1989), phonemic awareness, using the Elision subtest from the Comprehensive Test of Phonological Processing (Wagner et al., 1999), and contextual reading using the passage comprehension subtest (Woodcock et al., 1989).

### Executive Functions and Emotional Measures

Using several subtests, the EF were mapped to their sub-domains: (1) Attention using speed and accuracy subtests from the TEA-Ch battery [Sky Search, Score! and Sky-search DT subtests] (Manly et al., 1999); (2) Verbal fluency using the Delis-Kaplan Executive Functions System [D-KEF battery (Dellis et al., 2001)]; (3) Speed of processing using the “object naming” subtest from the CTOPP (Wagner et al., 1999); (4) Switching using the Wisconsin Card Sorting Task (Nyhus and Barceló, 2009); (5) Inhibition using the Stroop subtests from the D-KEFS (Dellis et al., 2001); and (6) Overall EF measured by the Behavior Rating Inventory of Executive Functions (BRIEF) (Gioia et al., 2000). Emotional abilities were assessed using the Emotional Control scale from the BRIEF (Nyhus and Barceló, 2009). Associations between reading, EF and emotional abilities were determined using Pearson correlations. Data was corrected using a Bonferroni correction.

### Neuroimaging Measures

#### Data Acquisition and Preprocessing

All images were acquired using a Philips Achieva 3T MRI scanner (Philips Medical Systems, Best, Netherlands). A T2^∗^-weighted, gradient-echo, echo planar imaging (EPI) sequence was used with fMRI parameters: TR/TE = 2000/38 msec, matrix size = 64 × 64, slice thickness = 5 mm, resulting in a voxel size = 4 × 4 × 5 mm^3^.

##### Resting-state task

Two five-minute resting-state scans were acquired, resulting in a total of 300 whole-brain volumes acquired in a total imaging time of 10 min. The initially acquired 10 time points were discarded to allow for T1 relaxation equilibrium. In addition, a high-resolution T1-weighted 3D anatomical scan was acquired using an inversion recovery (IR)-prepared turbo gradient-echo acquisition protocol with a spatial resolution of 1 × 1 × 1 mm^3^. Participants were acclimated and desensitized to the scanner to condition them for comfort during imaging (Vannest et al., 2014) and were instructed to keep their eyes open and to look at a cross on the screen during the resting state condition.

#### Functional Connectivity Analysis

**FIGURE 1:**
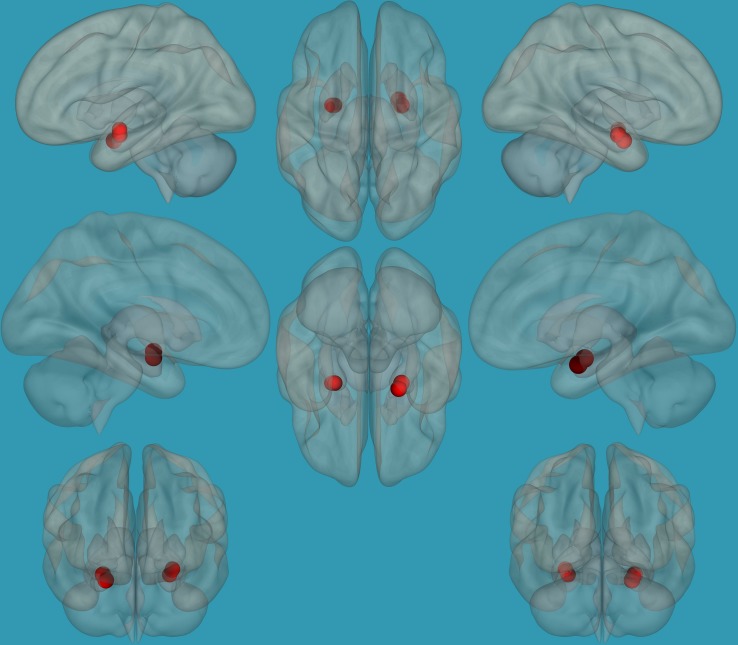
Seeds for the amygdala network. The seeds for the amygdala network: sagittal, axial, and coronal axis. The red color represents the seeds. Neurological orientation (L, **left**; R, **right**).

Regions for the emotional network were based on the amygdala seeds and were defined based on the literature (see Table 1). Functional-connectivity analysis during resting state was carried out using the CONN toolbox (Whitfield-Gabrieli and Nieto-Castanon, 2012). Normalized bias-corrected T_1_ images were generated in SPM^1^ and segmented into gray matter, white matter, and cerebral spinal fluid (CSF). The principle eigenvariate of the BOLD time-courses from the white matter and CSF, as well as the six motion-correction parameters, were included as regressors of no interest and removed from the fMRI time-series data. The data was band-pass filtered between 0.008 and 0.2 Hz [as recommended (Baria et al., 2011)], and only data within this range was included in the analysis. Networks were defined according to the regions of interest (ROIs) listed in Table 1 and shown in Figure 1, by equally weighting each of the ROIs within each network. Then, several functional connectivity analyses were performed: functional-connectivity within the amygdala and between the amygdala and other regions in the brain during rest in children with RD and in typical readers.

**TABLE 1:** Regions of interest in the emotional network for the current analyses [based on Roy et al. (2009)].

Specific site	X	Y	Z
Amygdala BLA (R)	22	−4	−22
Amygdala CAN (R)	24	−8	−18
Amygdala BLA (L)	−26	−9	−14
Amygdala CAN (L)	−24	−8	−18

*BLA, basolateral amygdaloidal complex; CAN, central amygdala nucleus; L, left; R, right; X/Y/Z, centroid coordinates.*

Functional connectivity on the network level (i.e., global efficiency) was calculated in CONN using a formula based on Latora and Marchiori (2001):

$$E=\frac{1}{n}\,\sum_{i\in N}E_{i}=\frac{1}{n}\,\sum_{i\in N}\frac{\sum_{j\in N,j\neq 1}d_{i\,j}^{-1}}{n-1}$$

where *E*_*i*_ is the efficiency of node *i*, *n* is the number of network nodes, *N* is the set of all network nodes, and *d${}_{i\,j}^{-1}$* is the inverse shortest path length between nodes *j* and *I*.

#### Seed-to-Voxel Analysis

To address the lateralization question, additional seed-to-voxel analyses were conducted. ROIs related to the amygdala (as implemented in the FSL Harvard-Oxford atlas in CONN) were divided into left and right ROIs, and each was defined as a seed in our analysis. We related to the left and right amygdala separately as seeds and correlated their BOLD response with the residual BOLD signal of each voxel in the brain. We then set a threshold for the resulting map of *p* = 0.05 voxel-height, false-discovery-rate (FDR) corrected for multiple comparisons and *p* = 0.05 cluster-size, FDR-corrected. To address the study questions related to laterality differences in the amygdala network, a seed-to-voxel analysis (functional-connectivity) between (1) the right amygdala, (2) the left amygdala, (3) the left vs. the right amygdala, and (4) the right vs. the left amygdala and all voxels in the brain for each group separately, as well as comparing children with RD and typical readers, was performed.

#### Correlation Analyses of Functional Connectivity of the Left and Right Amygdala and Reading, Emotional, and EF Abilities

To assess the relationship between EF abilities as measured by the BRIEF (Gioia et al., 2000), a seed-to-voxel analysis was performed over a range of voxels that spans a greater extent of functionally relevant areas. After determining the left and right amygdala seed-to-voxel functional connectivity matrix, we performed a seed-to-voxel correlation analysis between the left and right amygdala with reading, emotional and EF abilities, with a voxel-height threshold of *p* = 0.05, FDR corrected.

#### Prediction of Reading, Emotional and EF Abilities Based on Functional Connections of the Left vs. Right Amygdala in Typical Readers and Children With RD

To determine if the difference between functional connectivity of the left and right amygdala in children with RD and typical readers predicts reading, emotional, and EF abilities, separate regression analyses were conducted using reading, the BRIEF emotional subtest, and the general EF score (from the BRIEF) tasks.

## Results

### Behavioral Results

Children with RD demonstrated significantly decreased reading (in all domains of reading), emotional, and EF abilities compared to typical readers (see Table 2 for details). In the current study, significant positive correlations were found between reading measures, EF and emotional measures across groups; i.e., decreased reading ability was related to decreased emotional and EF abilities. Emotional and EF abilities also showed positive correlations, whereby better emotional abilities were related to better EF in both groups (see Supplementary Material – Table 1 for details).

**TABLE 2:** Baseline behavioral, reading measure, cognitive control and emotional test scores for children with RD and typical readers.

Measure	Description (test)	Children with RD (A)	Typical Readers (B)	t-test P-value	Contrast
*General Ability*	General non-verbal intelligence (Toni, Standard Score)	97.57 (11.15)	101.57 (14.09)	1.22 (ns)	A < B
	General verbal ability (PPVT, Standard Score)	102.48 (12.52)	110.89 (21.65)	1.81 (ns)	A < B
*Attention*	Visual attention accuracy (Number of Correct items, TEA-Ch, Sky Search, Scaled Score)	9.67 (3.42)	11.03 (2.52)	1.83 (ns)	A < B
*Reading*	Phonemic awareness (Ellison subset, CTOPP, Scaled Score)	7.7 (2.32)	11.3 (2.43)	5.96 (***)	A < B
	Words reading efficiency (SWE subset, TOWRE, Percentile)	23.52 (25.25)	65.65 (23.09)	6.93 (***)	A < B
	Pseudo-words reading efficiency (PDE subset, TOWRE, Scaled Score)	83.52 (13.12)	108.84 (9.75)	8.86 (***)	A < B
	Non-timed decoding of word reading (Letter-Word, Woodcock-Johnson, Standard Score)	89.93 (14.85)	112.65 (9.61)	7.43 (***)	A < B
	Non-timed reading comprehension (Passage comprehension, Woodcock-Johnson, Scaled Score)	84.41 (14.49)	103.57 (6.76)	7.07 (***)	A < B
	Non-timed decoding of pseudo-words reading (Word-Attack subtest, Woodcock-Johnson, Standard Score)	93.67 (9.54)	108.86 (8.6)	6.67 (***)	A < B
*Cognitive Control*	Speed of processing: Numbers (Number Naming, CTOPP, Scaled Score)	8.63 (2.9)	10.93 (2.14)	3.65 (***)	A < B
	Speed of processing: Words (Letter Naming, CTOPP, Scaled Score)	8.04 (2.78)	9.98 (2.36)	3.02 (**)	A < B
	Inhibition abilities: STROOP (Color Word Condition, Time, D-KEFS, Standard Score)	9.7 (2.37)	11.62 (2.23)	3.31 (**)	A < B
	Inhibition abilities: Stroop (Color Word Condition, Corrected Errors, D-KEFS, Standard Score)	37.93 (31.93)	54.05 (26.97)	2.19 (*)	A < B
	Working memory (Digit Span, WISC, Standard Score)	8.41 (2.21)	10.12 (2.04)	3.2 (**)	A < B
	Working memory (Digit Span, WISC, Forward-last-attempted)	5.37 (1.28)	6.54 (1.41)	3.42 (**)	A < B
	Working memory (Digit Span, WISC, Backward-last-attempted)	3.67 (1.04)	4.51 (1.02)	3.26 (**)	A < B
	Information speed of processing (Coding, WISC, Standard Score)	9.11 (2.78)	10.97 (4.21)	2 (ns)	A < B
	Information speed of processing (Symbol Search, WISC, Standard Score)	11.19 (1.96)	11.81 (2.04)	1.23 (ns)	A < B
	Inhibition abilities: Stroop (Color naming Time, D-KEFS, Standard Score)	8.81 (3.43)	11.95 (2.74)	4.06 (***)	A < B
	Inhibition abilities: Stroop (Color Word naming Time, D-KEFS, Standard Score)	9.22 (2.98)	11.84 (2.39)	3.9 (***)	A < B
	Visual attention speed (Time Per Target, TEA-Ch Sky Search, Scaled Score)	11.89 (17.93)	14.92 (17.03)	0.69 (ns)	A < B
	Visual attention accuracy (Attention, TEA-Ch Sky Search, Scaled Score)	7.67 (2.96)	9.05 (2.61)	1.98 (ns)	A < B
	Switching abilities (Perseverative Error Percent, Wisconsin, T score)	7.67 (2.9)	9.35 (2.86)	2.32 (*)	A < B
	Switching abilities (Non-perseverative Error Percent, Wisconsin, T score)	51.45 (11)	57.29 (9.37)	2.29 (*)	A < B
	Switching abilities (Categories Completed, Wisconsin, Percentile)	50.77 (12.54)	56.33 (11.06)	1.88 (ns)	A < B
	Switching abilities (Learning to Learn, Wisconsin, Percentile)	14.47 (6.3)	15.96 (5.99)	0.96 (ns)	A < B
	Switching abilities (Failure to maintain set, Wisconsin, Percentile)	13.06 (4.87)	16 (0)	3.68 (***)	A < B
	BRIEF Questionnaire: Executive Functions (Inhibit, BRIEF parent report, T Score)	14.92 (2.47)	13.82 (4.6)	−1.13 (ns)	B < A
	BRIEF Questionnaire: Executive Functions (Shift, BRIEF parent report, T Score)	53.41 (12.44)	46.97 (9.11)	−2.39 (*)	B < A
	BRIEF Questionnaire: Executive Functions (Initiate, BRIEF parent report, T Score)	53.48 (12.97)	47.73 (10.24)	−1.98 (ns)	B < A
	BRIEF Questionnaire: Executive Functions (Working Memory, BRIEF parent report, T score)	53.26 (11.38)	46.92 (8.48)	−2.55 (*)	B < A
	BRIEF Questionnaire: Executive Functions (Plan Organize, BRIEF parent report, T Score)	55.97 (11.71)	45.51 (9.47)	−3.95 (***)	B < A
	BRIEF Questionnaire: Executive Functions (Monitor, BRIEF parent report, T score)	57.66 (10.86)	44.84 (8.13)	−5.41 (***)	B < A
	BRIEF Questionnaire: Executive Functions (GEC, BRIEF parent report, T score)	55.35 (10.61)	45.46 (6.68)	−4.57 (***)	B < A
*Emotion-al ability*	BRIEF Questionnaire: Emotional Score (Emotional, BRIEF parent report, T score)	56.29 (11.43)	45.22 (9.47)	−4.23 (***)	B < A

RD, reading difficulties; TR, typical readers. Results are presented as mean (standard deviation). *p < 0.05; **p < 0.01; ***p < 0.001.

### Neuroimaging Results

#### Differences in Global Efficiency of the Amygdala Between Children With RD and Typical Readers

Global efficiency values of the amygdala network were defined (*p* < 0.05, FDR corrected for multiple comparisons), following which a two-sample *t*-test analysis was conducted for global efficiency within the amygdala network. The analysis revealed that children with RD showed significantly lower global efficiency values within the amygdala network compared to typical readers (children with RD: mean = 0.08, SD = 0.001, typical readers: mean = 1, SD = 0.02, *t* = 290.442, *p* < 0.01).

#### Correlation Between the Global Efficiency of the Amygdala and EF and Emotional Measures

Pearson correlations between the global efficiency of the amygdala (see Table 1 for the regions comprising the amygdala) and emotional and EF behavioral measures revealed significant negative correlations between the global efficiency of the amygdala network and EF abilities (with the emotional BRIEF sub-test: [*r* = −0.248, *p* < 0.05]; the EF BRIEF sub-test: working memory [*r* = −0.451, *p* < 0.01]; planning and organizing [*r* = −0.571, *p* < 0.01], and monitoring [*r* = −0.506, *p* < 0.01]). Increased functional connectivity within this network was associated with increased emotional ability, and lower BRIEF scores across groups. Increased functional connectivity within this network was associated with increased EF abilities (lower BRIEF scores) in both groups.

#### Differences in Functional Connectivity of the Amygdala Within Each Group Separately: Seed-to-Voxel Analysis

**FIGURE 2:**
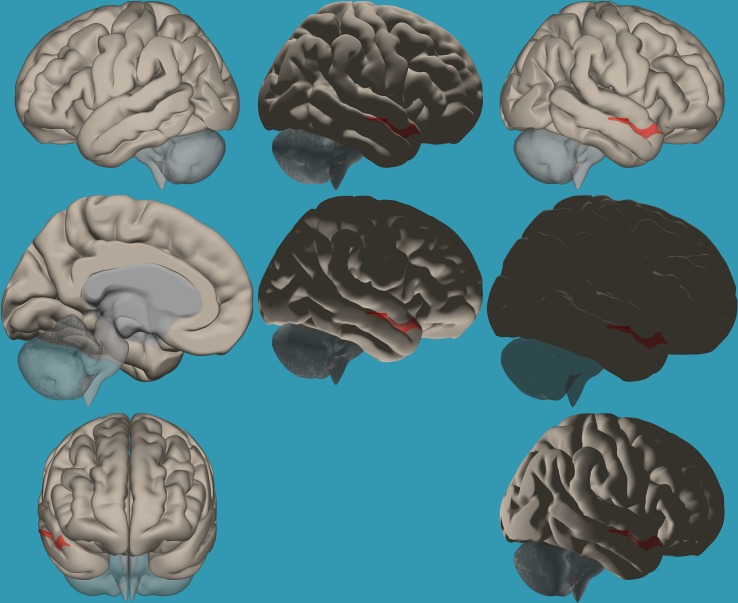
Seed-voxel functional connectivity between the right vs. left amygdala and all voxels in the brain in typical readers. Seed-to-voxel analysis for typical readers, right amygdala > left amygdala. Hot colors represent voxels with higher connectivity values (*p* < 0.05, FDR corrected). Neurological orientation (L, **left**; R, **right**). The colors in the figures do not represent the level of functional connections (a specific scale) but the existence of functional connections (all or none). This applies to all figures.

**FIGURE 3:**
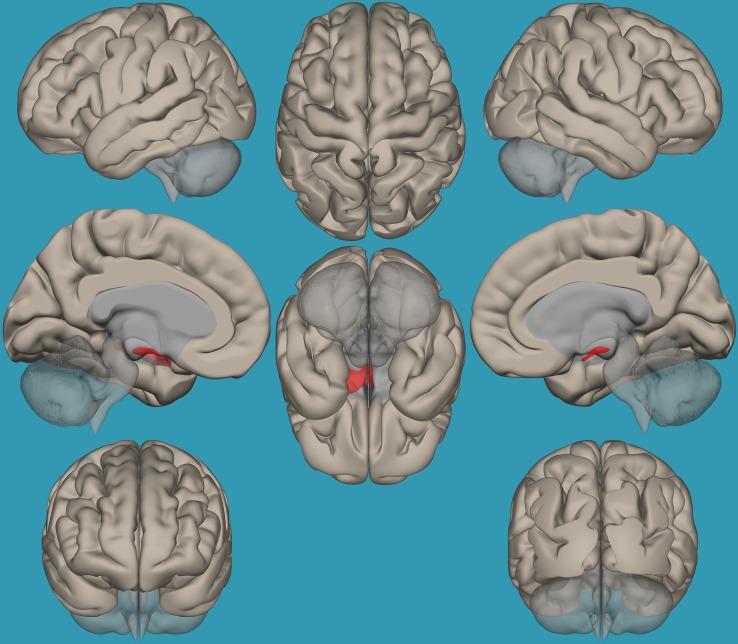
Seed-voxel functional connectivity between the left vs. right amygdala and all the voxels in the brain in typical readers. Seed-to-voxel analysis for typical readers, left amygdala > right amygdala. Hot colors represent voxels with higher connectivity values (*p* < 0.05, FDR corrected). Neurological orientation (L, **left**; R, **right**).

**FIGURE 4:**
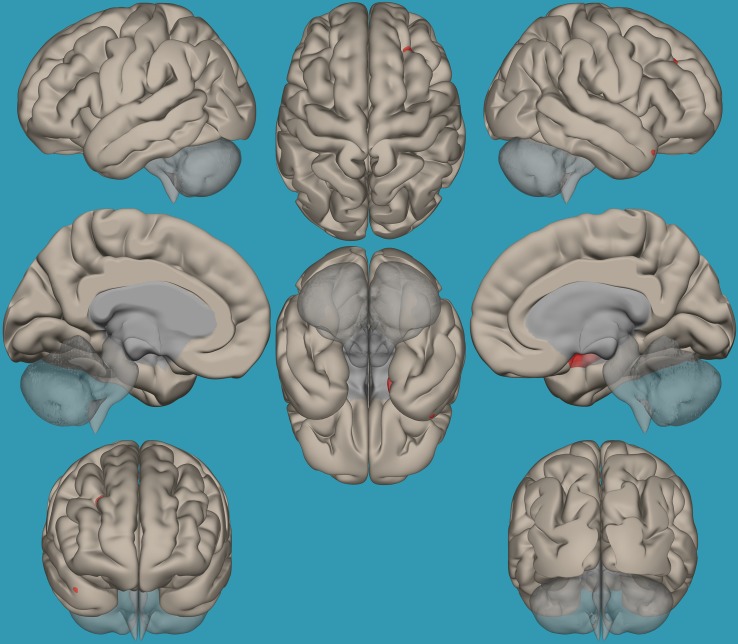
Seed-voxel functional connectivity between the right vs. left amygdala and all the voxels in the brain in children with reading difficulties (RD). Seed-to-voxel analysis for children with RD, right amygdala > left amygdala. Hot colors represent voxels with higher connectivity values (*p* < 0.05, FDR corrected). Neurological orientation (L, **left**; R, **right**).

**FIGURE 5:**
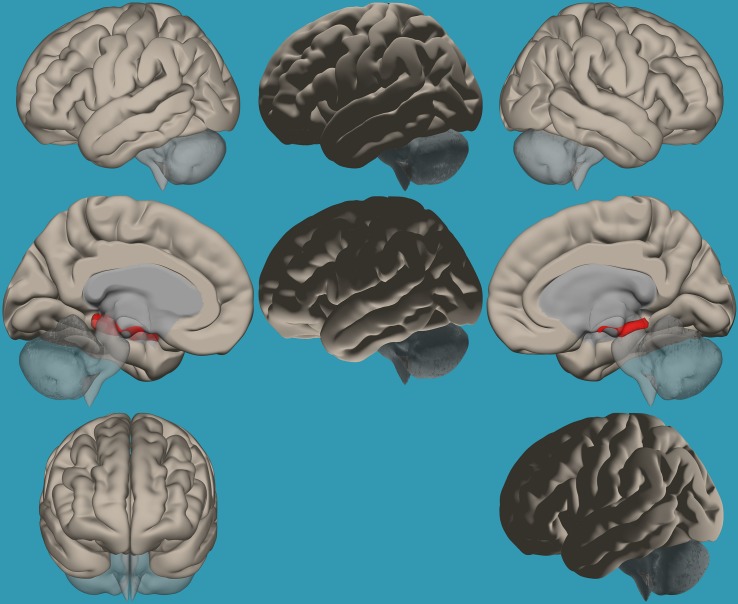
Seed-voxel functional connectivity between the left vs. right amygdala and all voxels in the brain in children with reading difficulties (RD). Seed-to-voxel analysis for children with RD, left amygdala > right amygdala. Hot colors represent voxels with higher connectivity values (*p* < 0.05, FDR corrected). Neurological orientation (L, **left**; R, **right**).

To establish functional connectivity of the right and left amygdala with the whole brain in each group separately, seed-to-voxel analysis was conducted (a voxel-height threshold of *p* = 0.05, FDR corrected). Voxel clusters that showed significant functional connectivity with the right and left amygdala in each group are presented in Table 2 in the Supplementary Material. Based on the results demonstrating the differences in functional connectivity within the amygdala between children with RD and typical readers, a two-sample *t*-test analysis comparing the functional connectivity between the two amygdala seeds (left vs. right amygdala) was conducted within each group separately (*p* < 0.05, FDR corrected). Typical readers demonstrated greater functional connectivity between the right vs. left amygdala and the right temporal pole. When the opposite contrast was compared (left vs. right amygdala), these readers demonstrated greater functional connectivity with the left insular cortex and left cingulate gyrus. Children with RD demonstrated greater functional connectivity of the right vs. left amygdala with the right temporal pole and the right middle frontal gyrus. These children also demonstrated a greater functional connectivity between the left vs. right amygdala and the left hippocampus; see Table 3 and Figures 2–5.

**TABLE 3:** Analyses (*t*-test) for the seed-voxel functional connectivity analysis for typical readers and children with reading difficulties; contrast: Left amygdala vs. right amygdala (*p* < 0.05, FDR corrected).

Group	Amygdala seed (contrast)	Clusters (anatomical regions)	Number of voxels in the cluster	X	Y	Z	Effect size (beta)	T
TR	R > L	Right temporal pole	5027	24	0	−16	0.15	14.9
TR	L > R	Left insular cortex	2671	−24	0	−16	0.16	15.6
		Left cingulate gyrus	317	−22	−16	44	0.07	4.97
RD	R > L	Right temporal pole	2201	26	0	−16	0.24	9.03
		Right middle frontal gyrus	267	28	32	26	0.11	4.64
RD	L > R	Left hippocampus	2937	−26	0	−18	0.21	7.88

*FDR, false discovery rate; TR, typical readers; RD, children with reading difficulties; L, left; R, right; X/Y/Z, centroid coordinates.*

#### Differences in Functional Connectivity of the Amygdala Between the Groups: Seed-to-Voxel Analysis

**FIGURE 6:**
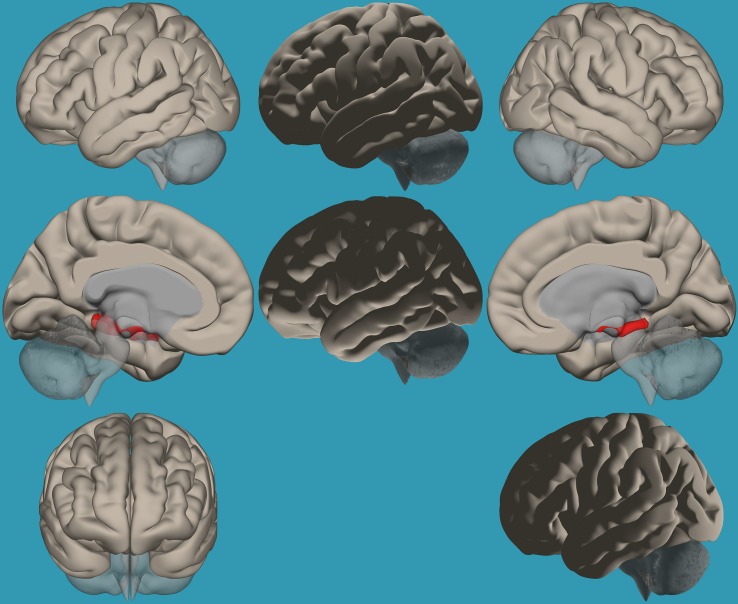
Seed-voxel functional connectivity between the left vs. right amygdala and all voxels in the brain in typical readers vs. children with reading difficulties (RD). Seed-to-voxel analysis for typical readers > children with RD, left amygdala > right amygdala. Hot colors represent voxels with higher connectivity values (*p* < 0.05, FDR corrected). Neurological orientation (L, **left**; R, **right**).

Based on the results demonstrating the differences in functional connectivity within the amygdala network between children with RD and typical readers, a two-sample *t*-test analysis comparing the functional connectivity between the two amygdala seeds (left vs. right) was conducted. Results revealed increased functional connectivity between the left vs. right amygdala and the right frontal pole in typical readers vs. children with RD (*p* < 0.05, FDR corrected); see Table 4 and Figure 6.

**TABLE 4:** Analyses (*t*-test) for the seed-voxel functional connectivity analysis for typical readers compared to children with reading difficulties (*p* < 0.05, FDR corrected).

Group contrast	Seed	Clusters (anatomical regions)	Number of voxels in the cluster	X	Y	Z	Effect size (beta)	T
TR > RD	L > R	Right frontal pole	336	26	38	28	0.12	4.49
TR > RD	L	Left frontal pole	324	−38	62	6	0.17	4.45
TR > RD	R	Left thalamus	1388	−12	0	10	0.12	5.59*

*P threshold of 0.01 uncorrected for multiple comparisons. FDR, false discovery rate; X/Y/Z, centroid coordinates; TR, typical readers; RD, children with reading difficulties.

**FIGURE 7:**
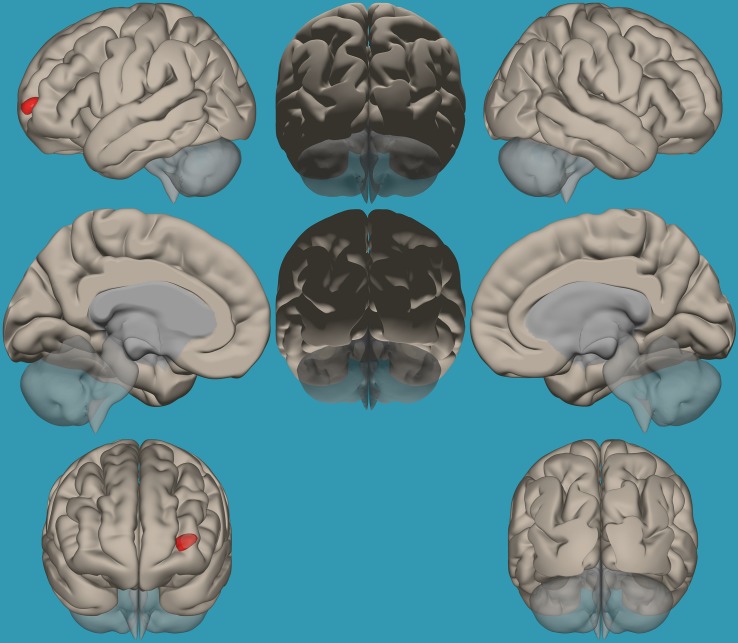
Seed-voxel functional connectivity between the left amygdala and all voxels in the brain in typical readers vs. children with reading difficulties (RD). Seed-to-voxel analysis for typical readers > children with RD, with the seed as the left amygdala. Hot colors represent voxels with higher connectivity values. Contrast: (*p* < 0.05, FDR corrected). Neurological orientation (L, **left**; R, **right**).

**FIGURE 8:**
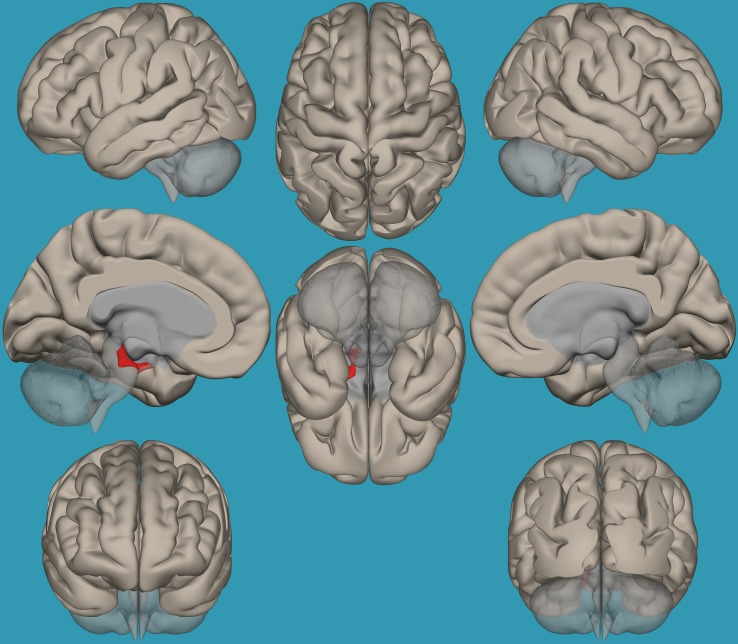
Seed-voxel functional connectivity between the right amygdala and all voxels in the brain in typical readers vs. children with reading difficulties (RD). Seed-to-voxel analysis for typical readers > children with RD, with the seed as the right amygdala. Hot colors represent voxels with higher connectivity values. Contrast (*p* < 0.01, FDR uncorrected). Neurological orientation (L, **left**; R, **right**).

To pinpoint the functional connections between the left amygdala, as the seed showing significant differences, and the whole brain in typical readers versus children with RD, two-way *t*-tests examining the functional connectivity between the left amygdala and the whole brain in both groups were conducted. Results revealed a significantly greater functional connectivity between the left amygdala and the left frontal pole in typical readers vs. children with RD (*p* < 0.05, FDR corrected) (Figure 7). Choosing the right amygdala as a seed and contrasting typical readers with children with RD did not reveal significant results within a threshold of 0.05 corrected for multiple comparisons. Lowering the threshold to *p* < 0.01 uncorrected for multiple comparisons revealed that typical readers demonstrated an increased functional connectivity between the right amygdala and the left thalamus compared to children with RD; see Table 4 and Figures 7, 8.

#### Pearson Correlation Between Functional Connectivity of the Amygdala and the Entire Brain (Seed-to-Voxel Analysis) and Behavioral Measures (Reading, Emotional and EF Abilities)

Due to the observed differences in functional connectivity of the right vs. left amygdala and the entire brain between typical readers and children with RD, Pearson correlation analyses between functional connectivity of the left and right amygdala separately and the entire brain, with several behavioral measures for reading, emotional, and EF abilities in the entire study population (children with RD and typical readers), were conducted. Positive correlation was found between reading measures (phonological processing abilities [CTOPP Ellison subset (*r* = 0.348, *p* < 0.01)], timed-word reading ability [TOWRE (*r* = 0.248, *p* < 0.05)], and untimed reading ability [Letter–Word (*r* = 0.446, *p* < 0.01)]) and functional connectivity of the left amygdala with both the left and right frontal poles. Negative correlation was found between emotional abilities and functional connectivity of the left amygdala with the right frontal pole [BRIEF emotional subtest (*r* = −0.314, *p* < 0.01)]. Positive correlation was found between EF measures and functional connectivity of the left amygdala with both the left and right frontal poles for switching abilities [Stroop Color Word Condition, Corrected Errors (*r* = 0.247, *p* < 0.05)], working memory [digit span test (*r* = 0.247, *p* < 0.05)], and learning from errors [Wisconsin Non-perseverative Error (*r* = 0.356, *p* < 0.01)]. Negative correlation was found between the BRIEF cognitive subtests and functional connectivity of the left amygdala with the right frontal pole using the BRIEF overall general score (*r* = −0.317, *p* < 0.01). Increased functional connectivity of the left amygdala and the whole brain was related to an increased reading ability, as well as emotional and EF performance, across all participants.

#### Regression Analyses Between Functional Connectivity Results and Reading, Emotional, and EF Abilities

To determine whether the functional connectivity in the left vs. right amygdala in typical readers vs. children with RD explains reading, emotional, and EF abilities in these groups, three separate regression analysis models were conducted.

##### Reading measures

A linear regression established that functional connectivity of the left vs. right amygdala to the right frontal pole and left amygdala to the left frontal pole significantly predicted passage comprehension subtest scores in both groups [*F*(1,62) = 7.73, *p* < 0.05] and accounted for 52% of the explained variability in passage comprehension scores. Increased functional connectivity between these regions was associated with higher reading scores.

##### Emotional abilities

A linear regression established that functional connectivity of the left vs. right amygdala to the right frontal pole significantly predicted emotional abilities in both groups [*F*(1,62) = 6.785, *p* < 0.05] and accounted for 31.4% of the explained variability in the BRIEF emotional scores. Higher functional connectivity between the left vs. right amygdala and the right frontal pole predicted lower scores in the BRIEF emotional subsets, reflecting less emotional difficulties within the entire population.

##### EF measures

A linear regression established that functional connectivity of the left vs. right amygdala to the right frontal pole and the left amygdala to the left frontal pole significantly predicted switching abilities (Wisconsin test switching abilities (Non-perseverative Error Percent) [*F*(1,62) = 13.02, *p* < 0.05] and accounted for 42% of the explained variability in switching abilities. Increased functional connectivity between these regions was associated with higher cognitive scores.

## Discussion

The current study was designed to reveal neurobiological evidence of the involvement of emotional difficulties in children with RD, focusing on amygdala laterality. We determined the existence of such neurobiological evidence by assessing the differences in functional connectivity within the amygdala, its left and right components, and the entire brain between children with RD and typical readers.

In addition to the decreased EF and emotional abilities previously found in children with RD and typical readers (Nachshon and Horowitz-Kraus, 2018), our results suggest that the global efficiency of the amygdala is decreased in those with RD compared to typical readers, and that the left and right amygdala contribute to reading, emotional, speed of processing and EF abilities in an unequal manner; i.e., greater functional connections between the left vs. right amygdala and frontal cortices were related to increased reading, emotional, speed of processing and EF abilities. Therefore, for the first time, we have demonstrated a linkage between the laterality of the amygdala functional connectivity and frontal regions and reading, emotional and EF abilities.

### Decreased Emotional Skills Are Associated With Decreased EF Abilities

We previously reported emotional difficulties in children with RD (Nachshon and Horowitz-Kraus, 2018), which supported other earlier findings (Grills-Taquechel et al., 2012). These difficulties are also found among children facing challenges in EF, and there exist correlations between these challenges and reading. This connection exemplifies the reading–EF–emotional triangle in children with RD. The behavioral reports of impaired emotional abilities in children with RD are supported by the current neuroimaging study. Our results indicate that children with RD showed a significantly lower global efficiency of the amygdala network, which included the left and right amygdala seeds, compared to typical readers. The global efficiency measure is related to the average inverse shortest path length to all other nodes in the network, which is related to better synergy between the seeds in the network (Rubinov, 2010). These results enhance previously reported results of decreased global efficiency in EF networks (cingulo-opercular) that was related to decreased reading abilities in children with RD (Horowitz-Kraus et al., 2015). Decreased global efficiency is related to the inefficiency of the network nodes to activate simultaneously, which may be a factor contributing to the emotional difficulties in children with RD. The fact that the neuroimaging condition used in the current study is a resting state condition, without any written stimuli, strengthens the assumption that the impairment in this emotional network among children with RD is not specific to their interaction with written stimuli, but can be generalized to non-reading conditions and everyday life experiences.

### Separate Roles for the Left and Right Amygdala

Our results confirm our hypothesis that children with RD would show different functional connectivity in the left vs. right amygdala compared to typical readers, which will be related to their behavioral performance. We found that children with RD demonstrated decreased functional connectivity between the left vs. right amygdala and the left and right frontal poles compared to typical readers, which was related to decreased reading, emotional, and EF abilities in this group. Since the difference between functional connectivity of the left and right amygdala and the entire brain was reduced in children with RD, we suggest that either overactivation of the right amygdala or decreased activity of the left amygdala in children with RD is driving these differences, compared to typical readers. The role of the right hemisphere in processing emotional stimuli was previously described by others. Morris et al. (1999) showed a significant role of the right amygdala seeds in processing of “unseen” fear stimuli, while left amygdala seeds did not show any change in connectivity. Davis et al. (2018) also showed an increased functional connectivity between the left basolateral amygdala and left mPFC, and between the bilateral centro-medial amygdala and left mPFC in children with RD, while decreased functional connectivity was observed in typical readers. In addition, they showed that functional connectivity of the right centro-medial amygdala and the left mPFC positively predicted anxiety symptoms, as was suggested in the current study. The role of the right amygdala, and specifically connectivity with prefrontal regions, was previously related to processing of subconscious fear stimuli (Morris et al., 1999).

The fact that no significant differences were found when comparing typical readers and children with RD on functional connectivity of the right hemisphere with all the voxels in the brain (i.e., results did not survive the correction for multiple comparisons and only showed results at *p* < 0.01, uncorrected for multiple comparisons), however, may rule out the assumption that the differences in lateralization between the left and right amygdala result from decreased right-lateralized connectivity. It does, nevertheless, strengthen the assumption that the differences stem from decreased functional connections of the left hemisphere with all the voxels in the brain in children with RD. Our results not only support a relationship between decreased EF abilities and functional connections of the right amygdala and frontal cortices, but also suggest that the left amygdala demonstrates greater functional connections with frontal regions compared to the right amygdala in children with RD. Moreover, the results suggest that these greater connections between the left vs. right amygdala in children with RD are related to their reading, emotional and EF performance. This may indicate that not only is the right amygdala processing emotional stimuli in an imprecise way in children with RD, but that the left amygdala, which would have been expected to have demonstrated greater functional connections than the right amygdala, does not demonstrate this pattern in children with RD.

An alternative explanation may be derived from reports showing overall increased activation in the right hemisphere in children with RD (Horowitz-Kraus and Breznitz, 2014). Increased right-lateralized activation in reading-related regions was reported in children with RD, with the authors of that study theorizing that this ineffective processing pattern may be one cause of the RD (Horowitz-Kraus and Breznitz, 2014). Based on our results, we suggest that children with RD may show a generally greater reliance on the right hemisphere, which was suggested previously as one reason for the increased artistic and creative abilities in this population (Wolff and Lundberg, 2002). The increased reliance on the right hemisphere may also be related to increased functional connections of the right amygdala in these readers compared to typical readers. Future research should explore this further and create a lateralization index for the right vs. left hemispheres in children with RD.

### Study Limitations

When reviewing the results of this study, the following limitations should be weighed. First, although the BRIEF questionnaire utilized 10 questions to cover the emotional abilities domain, only one emotional subtest was used in this study, compared to a variety of subtests used for EF and reading. Nevertheless, it is important to note that the test used covers a variety of domains, such as emotional control. A future study should collect additional emotional measures, e.g., for anxiety, self-efficacy, self-esteem, and social behavior. A second limitation is related to not yet determining whether emotional difficulty is the source of reading impairment or if the reverse is the case. A longitudinal research study may reveal the origins of the variety of difficulties in children with RD. Third, the current study used resting state data to examine the association between the amygdala functional connectivity and reading and EF. Future research should include a specific emotional-based paradigm to examine the emotional network and its association with reading and EF.

## Conclusion

The results of this study demonstrated that children with RD also suffer from lower emotional skills that conjoin with impairments in both reading and EF, and strengthen the role of the amygdala in controlling aspects of their EF and reading abilities. Both global efficiency of the amygdala and functional connectivity of the left vs. right amygdala to frontal regions predicted and explained differences in EF and reading abilities, which highlight the amygdala as a critical region of the reading network to be considered in future studies (Stanislas, 2009).

## Data Availability Statement

The raw data supporting the conclusions of this article will be made available by the authors, without undue reservation.

## Ethics Statement

This study was carried out in accordance with the recommendations of Cincinnati Children’s Hospital review board with written informed assent signed by participants and informed consent signed by parents in accordance with the Declaration of Helsinki. The protocol was approved by the CCHMC IRB Committee.

## Author Contributions

ON: data analysis, interpretation, manuscript writing, and approving final version. TH-K: study design, data acquisition, analysis, interpretation, manuscript writing and revising, and approving final version for publication. RF: data analysis and interpretation, manuscript writing and revising, and approving final version for publication.

## Conflict of Interest

The authors declare that the research was conducted in the absence of any commercial or financial relationships that could be construed as a potential conflict of interest.

## Footnotes

1 http://www.fil.ion.ucl.ac.uk/spm/

## Supplementary Material

TABLE S1 Pearson correlations between emotional, reading and EF abilities in the typical readers group and in the reading difficulties group.

TABLE S2 Voxel clusters showing significant functional connectivity with the right and left amygdala in each group.
