# Supplementary material for: Decreased Functional Connectivity Between the Left Amygdala and Frontal Regions Interferes With Reading, Emotional, and Executive Functions in Children With Reading Difficulties
Source: Front Hum Neurosci. 2020 Apr 28;14:104. doi: 10.3389/fnhum.2020.00104 (PMC7198704; doi:10.3389/fnhum.2020.00104)
Supplement: TABLE S2 — Voxel clusters showing significant functional connectivity with the right and left amygdala in each group. [file Table_2.docx]

**Supplemental Table 2. Voxel clusters showing connectivity between the left and right Amygdala and the entire brain in typical readers and children with reading difficulties**

Voxel clusters showing connectivity between the left Amygdala and the entire brain typical readers (TRs)

| Voxel cluster count | Region |
| --- | --- |
| 3284 | Precentral Gyrus Left |
| 3193 | Frontal Pole Left |
| 3034 | Frontal Pole Right |
| 2875 | Lateral Occipital Cortex, superior division Left |
| 2681 | Postcentral Gyrus Left |
| 2483 | Precentral Gyrus Right |
| 2322 | Cingulate Gyrus, anterior division |
| 2113 | Precuneous Cortex |
| 2092 | Postcentral Gyrus Right |
| 2041 | Lateral Occipital Cortex, inferior division Left |
| 1970 | Brain stem |
| 1898 | Occipital Pole Left |
| 1823 | Temporal Pole Left |
| 1738 | Temporal Pole Right |
| 1715 | Lateral Occipital Cortex, inferior division Right |
| 1590 | Frontal Orbital Cortex Left |
| 1410 | Cerebelum 6 Right |
| 1391 | Occipital Pole Right |
| 1345 | Frontal Orbital Cortex Right |
| 1344 | Insular Cortex Right |
| 1334 | Insular Cortex Left |
| 1308 | Lingual Gyrus Left |
| 1302 | Lingual Gyrus Right |
| 1265 | Cerebelum 6 Left |
| 1127 | Cerebelum Crus1 Right |
| 1066 | Paracingulate Gyrus Right |
| 1064 | Cerebelum Crus1 Left |
| 1028 | Paracingulate Gyrus Left |
| 1021 | Middle Temporal Gyrus, temporooccipital part Right |
| 977 | Central Opercular Cortex Left |
| 912 | Middle Temporal Gyrus, posterior division Left |
| 905 | Thalamus |
| 883 | Thalamus |
| 869 | Central Opercular Cortex Right |
| 867 | Putamen |
| 863 | Middle Temporal Gyrus, temporooccipital part Left |
| 834 | Lateral Occipital Cortex, superior division Right |
| 813 | Middle Temporal Gyrus, posterior division Right |
| 804 | Putamen |
| 784 | Occipital Fusiform Gyrus Left |
| 758 | Temporal Occipital Fusiform Cortex Right |
| 742 | Subcallosal Cortex |
| 735 | Supramarginal Gyrus, anterior division Left |
| 732 | oper l Inferior Frontal Gyrus, pars opercularis |
| 696 | Hippocampus |
| 691 | Temporal Fusiform Cortex, posterior division Left |
| 673 | oper r Inferior Frontal Gyrus, pars opercularis |
| 660 | Frontal Medial Cortex |
| 644 | Temporal Occipital Fusiform Cortex Left |
| 636 | Inferior Temporal Gyrus, temporooccipital part Left |
| 631 | Hippocampus |
| 605 | l Inferior Frontal Gyrus, pars triangularis Left |
| 605 | Occipital Fusiform Gyrus Right |
| 599 | Cerebelum 4 5 Right |
| 588 | Intracalcarine Cortex Left |
| 582 | Supramarginal Gyrus, posterior division Right |
| 575 | Supramarginal Gyrus, anterior division Right |
| 563 | Planum Temporale Left |
| 558 | Parietal Operculum Cortex Left |
| 558 | Vermis 4 5 |
| 547 | r Inferior Frontal Gyrus, pars triangularis Right |
| 535 | Parahippocampal Gyrus, anterior division Left |
| 527 | Parietal Operculum Cortex Right |
| 527 | Cerebelum Crus2 Right |
| 519 | Parahippocampal Gyrus, anterior division Right |
| 505 | Caudate |
| 502 | Middle Frontal Gyrus Left |
| 496 | Cingulate Gyrus, posterior division |
| 495 | Inferior Temporal Gyrus, temporooccipital part Right |
| 494 | Cuneal Cortex Left |
| 493 | Temporal Fusiform Cortex, posterior division Right |
| 487 | Supramarginal Gyrus, posterior division Left |
| 460 | Caudate |
| 437 | Planum Temporale Right |
| 432 | Cuneal Cortex Right |
| 424 | Middle Temporal Gyrus, anterior division Left |
| 390 | Parahippocampal Gyrus, posterior division Left |
| 388 | Angular Gyrus Left |
| 384 | Superior Frontal Gyrus Left |
| 383 | Superior Temporal Gyrus, posterior division Left |
| 377 | Planum Polare Right |
| 365 | Middle Temporal Gyrus, anterior division Right |
| 363 | Superior Temporal Gyrus, posterior division Right |
| 362 | Cerebelum 9 Left |
| 358 | Planum Polare Left |
| 355 | Frontal Operculum Cortex Left |
| 342 | Amygdala |
| 336 | Cerebelum 8 Right |
| 331 | Vermis 6 |
| 327 | Amygdala |
| 319 | Parahippocampal Gyrus, posterior division Right |
| 313 | Frontal Operculum Cortex Right |
| 309 | Heschl's Gyrus Left |
| 282 | Heschl's Gyrus Right |
| 280 | Superior Temporal Gyrus, anterior division Left |
| 278 | Superior Temporal Gyrus, anterior division Right |
| 271 | Cerebelum 8 Left |
| 269 | Pallidum |
| 266 | Superior Frontal Gyrus Right |
| 259 | Pallidum |
| 213 | Vermis 3 |
| 209 | Angular Gyrus Right |
| 191 | Vermis 7 |
| 182 | Cerebelum 3 Right |
| 179 | Vermis 8 |
| 151 | Middle Frontal Gyrus Right |
| 135 | Superior Parietal Lobule Left |
| 132 | Cerebelum 3 Left |
| 129 | Cerebelum Crus2 Left |
| 112 | Cerebelum 9 Right |
| 111 | Inferior Temporal Gyrus, posterior division Left |
| 107 | Accumbens |
| 103 | Inferior Temporal Gyrus, anterior division Left |
| 91 | Superior Parietal Lobule Right |
| 85 | Inferior Temporal Gyrus, anterior division Right |
| 84 | Accumbens |
| 80 | Supracalcarine Cortex Right |
| 77 | Temporal Fusiform Cortex, anterior division Left |
|  |  |

Voxel clusters showing connectivity between the right Amygdala and the entire brain in typical readers (TRs)

| Voxel cluster count | Region |
| --- | --- |
| 3767 | Frontal Pole Right |
| 2835 | Precentral Gyrus Right |
| 2817 | Precentral Gyrus Left |
| 2486 | Postcentral Gyrus Left |
| 2469 | Postcentral Gyrus Right |
| 2279 | Frontal Pole Left |
| 2266 | Occipital Pole Left |
| 2208 | Cingulate Gyrus, anterior division |
| 2154 | Precuneous Cortex |
| 2099 | Lateral Occipital Cortex, superior division Left |
| 2040 | Brain stem |
| 2017 | Lateral Occipital Cortex, inferior division Left |
| 1904 | Occipital Pole Right |
| 1781 | Temporal Pole Right |
| 1724 | Lateral Occipital Cortex, inferior division Right |
| 1633 | Temporal Pole Left |
| 1589 | Frontal Orbital Cortex Left |
| 1496 | Lateral Occipital Cortex, superior division Right |
| 1399 | Lingual Gyrus Right |
| 1396 | Cerebelum 6 Right |
| 1390 | Frontal Orbital Cortex Right |
| 1374 | Lingual Gyrus Left |
| 1344 | Insular Cortex Right |
| 1334 | Insular Cortex Left |
| 1212 | Cerebelum 6 Left |
| 1123 | Thalamus |
| 1120 | Middle Temporal Gyrus, posterior division Right |
| 1119 | Paracingulate Gyrus Right |
| 1063 | Middle Temporal Gyrus, temporooccipital part Right |
| 1042 | Cerebelum Crus1 Right |
| 1024 | Paracingulate Gyrus Left |
| 970 | Central Opercular Cortex Left |
| 956 | Thalamus |
| 882 | Cerebelum 4 5 Left |
| 874 | Central Opercular Cortex Right |
| 867 | Putamen |
| 824 | Cerebelum Crus1 Left |
| 804 | Putamen |
| 800 | Occipital Fusiform Gyrus Left |
| 734 | Middle Temporal Gyrus, temporooccipital part Left |
| 723 | Temporal Occipital Fusiform Cortex Right |
| 712 | Hippocampus |
| 690 | Frontal Medial Cortex |
| 683 | Cingulate Gyrus, posterior division |
| 676 | Inferior Frontal Gyrus, pars opercularis Right |
| 675 | Inferior Frontal Gyrus, pars opercularis Left |
| 672 | Occipital Fusiform Gyrus Right |
| 668 | Subcallosal Cortex |
| 650 | Middle Temporal Gyrus, posterior division Left |
| 650 | Hippocampus |
| 640 | Temporal Occipital Fusiform Cortex Left |
| 629 | Temporal Fusiform Cortex, posterior division Left |
| 603 | l Inferior Frontal Gyrus, pars triangularis |
| 603 | Supramarginal Gyrus, anterior division Left |
| 600 | Vermis 4 5 |
| 598 | Cerebelum 4 5 Right |
| 590 | Superior Frontal Gyrus Right |
| 589 | Cerebelum 8 Right |
| 586 | Inferior Temporal Gyrus, temporooccipital part Left |
| 582 | Supramarginal Gyrus, posterior division Right |
| 577 | Intracalcarine Cortex Right |
| 574 | Supramarginal Gyrus, anterior division Right |
| 565 | Planum Temporale Left |
| 555 | Parietal Operculum Cortex Left |
| 553 | Inferior Frontal Gyrus, pars triangularis Right |
| 538 | Intracalcarine Cortex Left |
| 534 | Parietal Operculum Cortex Right |
| 529 | Parahippocampal Gyrus, anterior division Right |
| 509 | Caudate |
| 488 | Cuneal Cortex Right |
| 484 | Caudate |
| 463 | Parahippocampal Gyrus, anterior division Left |
| 462 | Cerebelum Crus2 Right |
| 447 | Temporal Fusiform Cortex, posterior division Right |
| 440 | Planum Temporale Right |
| 406 | Middle Temporal Gyrus, anterior division Left |
| 396 | Angular Gyrus Right |
| 395 | Superior Temporal Gyrus, posterior division Right |
| 393 | Supramarginal Gyrus, posterior division Left |
| 390 | Parahippocampal Gyrus, posterior division Left |
| 382 | Middle Temporal Gyrus, anterior division Right |
| 377 | Planum Polare Right |
| 370 | Inferior Temporal Gyrus, temporooccipital part Right |
| 361 | Superior Frontal Gyrus Left |
| 360 | Cuneal Cortex Left |
| 359 | Superior Temporal Gyrus, posterior division Left |
| 358 | Planum Polare Left |
| 344 | Frontal Operculum Cortex Left |
| 342 | Amygdala |
| 334 | Vermis |
| 332 | Middle Frontal Gyrus Left |
| 327 | Amygdala |
| 319 | Parahippocampal Gyrus, posterior division Right |
| 309 | Heschl's Gyrus Left |
| 307 | Frontal Operculum Cortex Right |
| 296 | Cerebelum 8 Left |
| 282 | Heschl's Gyrus Right |
| 280 | Superior Temporal Gyrus, anterior division Left |
| 278 | Superior Temporal Gyrus, anterior division Right |
| 277 | Middle Frontal Gyrus Right |
| 269 | Pallidum |
| 267 | Angular Gyrus Left |
| 262 | Cerebelum 9 Left |
| 259 | Pallidum |
| 228 | Vermis |
| 216 | Vermis |
| 212 | Cerebelum 9 Right |
| 186 | Vermis |
| 185 | Superior Parietal Lobule Right |
| 182 | Cerebelum 3 Right |
| 139 | Cerebelum Crus2 Left |
| 133 | Inferior Temporal Gyrus, anterior division Right |
| 132 | Cerebelum 3 Left |
| 124 | Supracalcarine Cortex Right |
| 107 | Accumbens |
| 97 | Temporal Fusiform Cortex, anterior division Right |
| 84 | Accumbens |
| 75 | Vermis |
| 68 | Inferior Temporal Gyrus, anterior division Left |
| 66 | Supracalcarine Cortex Left |
| 63 | Inferior Temporal Gyrus, posterior division Left |
| 57 | Cerebelum 7b Right |
| 54 | Temporal Fusiform Cortex, anterior division Left |

Voxel clusters showing connectivity between the left Amygdala and the entire brain in children with reading difficulties (RD)

| Voxel cluster count | Region |
| --- | --- |
| 2076 | Postcentral Gyrus Left |
| 1897 | Precentral Gyrus Left |
| 1709 | Postcentral Gyrus Right |
| 1584 | Brain stem |
| 1559 | Frontal Orbital Cortex Left |
| 1470 | Precentral Gyrus Right |
| 1462 | Temporal Pole Left |
| 1351 | Temporal Pole Right |
| 1318 | Cingulate Gyrus, anterior division |
| 1304 | Frontal Orbital Cortex Right |
| 1293 | Insular Cortex Right |
| 1251 | Insular Cortex Left |
| 1056 | Lingual Gyrus Right |
| 996 | Lingual Gyrus Left |
| 951 | Cerebelum 6 Left |
| 938 | Superior Parietal Lobule Right |
| 898 | Central Opercular Cortex Left |
| 867 | Frontal Pole Right |
| 815 | Cerebelum 4 5 Left |
| 813 | Cerebelum 6 Right |
| 785 | Putamen |
| 780 | Precuneous Cortex |
| 770 | Central Opercular Cortex Right |
| 763 | Putamen |
| 736 | Lateral Occipital Cortex, inferior division Right |
| 708 | Hippocampus |
| 679 | Middle Temporal Gyrus, temporooccipital part Right |
| 632 | Temporal Occipital Fusiform Cortex Left |
| 603 | Hippocampus |
| 589 | Subcallosal Cortex |
| 565 | Planum Temporale Left |
| 560 | Cerebelum 4 5 Right |
| 552 | Vermis 5 |
| 532 | Superior Parietal Lobule Left |
| 527 | Parietal Operculum Cortex Left |
| 478 | Temporal Occipital Fusiform Cortex Right |
| 477 | Parietal Operculum Cortex Right |
| 440 | Planum Temporale Right |
| 433 | Middle Temporal Gyrus, temporooccipital part Left |
| 416 | Inferior Frontal Gyrus, pars opercularis Right |
| 404 | Parahippocampal Gyrus, anterior division Left |
| 399 | Frontal Pole Left |
| 389 | Inferior Frontal Gyrus, pars triangularis Right |
| 385 | Parahippocampal Gyrus, posterior division Left |
| 376 | Planum Polare Right |
| 373 | Inferior Frontal Gyrus, pars opercularis Left |
| 361 | Parahippocampal Gyrus, anterior division Right |
| 358 | Temporal Fusiform Cortex, posterior division Left |
| 358 | Planum Polare Left |
| 350 | Supramarginal Gyrus, anterior division Left |
| 350 | Lateral Occipital Cortex, inferior division Left |
| 343 | Supramarginal Gyrus, posterior division Right |
| 342 | Amygdala |
| 341 | Middle Temporal Gyrus, posterior division Right |
| 340 | Superior Temporal Gyrus, posterior division Left |
| 338 | Supramarginal Gyrus, posterior division Left |
| 327 | Amygdala |
| 313 | Parahippocampal Gyrus, posterior division Right |
| 309 | Heschl's Gyrus Left |
| 305 | Supramarginal Gyrus, anterior division Right |
| 303 | Vermis |
| 282 | Heschl's Gyrus Right |
| 280 | Superior Temporal Gyrus, anterior division Left |
| 278 | Superior Temporal Gyrus, anterior division Right |
| 277 | Middle Temporal Gyrus, posterior division Left |
| 275 | Superior Temporal Gyrus, posterior division Right |
| 274 | Middle Temporal Gyrus, anterior division Left |
| 266 | Frontal Operculum Cortex Left |
| 253 | Pallidum |
| 238 | Frontal Operculum Cortex Right |
| 227 | Vermis |
| 221 | Temporal Fusiform Cortex, posterior division Right |
| 216 | Cingulate Gyrus, posterior division |
| 213 | Middle Temporal Gyrus, anterior division Right |
| 210 | l Inferior Frontal Gyrus, pars triangularis |
| 200 | Occipital Fusiform Gyrus Left |
| 189 | Angular Gyrus Right |
| 175 | Occipital Pole Right |
| 172 | Cerebelum 3 Right |
| 160 | Pallidum |
| 158 | Paracingulate Gyrus Left |
| 142 | Lateral Occipital Cortex, superior division Right |
| 133 | Cerebelum Crus1 Left |
| 132 | Frontal Medial Cortex |
| 131 | Cerebelum 3 Left |
| 123 | Angular Gyrus Left |
| 120 | Paracingulate Gyrus Right |
| 119 | Inferior Temporal Gyrus, temporooccipital part Right |
| 114 | Caudate |
| 110 | Thalamus |
| 106 | Accumbens |
| 99 | Inferior Temporal Gyrus, temporooccipital part Left |
| 91 | Lateral Occipital Cortex, superior division Left |
| 91 | Intracalcarine Cortex Left |
| 90 | Superior Frontal Gyrus Right |
| 88 | Thalamus |
| 85 | Caudate |
| 78 | Accumbens |
| 74 | Cerebelum 8 Right |
| 62 | Occipital Pole Left |
| 61 | Temporal Fusiform Cortex, anterior division Left |
| 58 | Vermis |
| 41 | Superior Frontal Gyrus Left |
| 41 | Intracalcarine Cortex Right |
| 41 | Cerebelum Crus1 Right |
| 37 | Cerebelum 9 Right |
| 24 | Cuneal Cortex Left |
| 20 | Cerebelum 9 Left |
| 20 | Vermis 2 |
|  |  |

Voxel clusters showing connectivity between the right Amygdala and the entire brain in children with reading difficulties (RD)

| Voxel cluster count | Region |
| --- | --- |
| 2365 | Postcentral Gyrus Right |
| 2156 | Postcentral Gyrus Left |
| 1891 | Temporal Pole Right |
| 1815 | Temporal Pole Left |
| 1615 | Frontal Pole Right |
| 1565 | Frontal Orbital Cortex Left |
| 1542 | Precentral Gyrus Right |
| 1450 | Cingulate Gyrus, anterior division |
| 1409 | Frontal Orbital Cortex Right |
| 1391 | Precentral Gyrus Left |
| 1364 | Lateral Occipital Cortex, inferior division Left |
| 1334 | Insular Cortex Right |
| 1288 | Insular Cortex Left |
| 1137 | Cerebelum Crus1 Left |
| 1130 | Lingual Gyrus Left |
| 1118 | Lateral Occipital Cortex, inferior division Right |
| 1078 | Lingual Gyrus Right |
| 1005 | Frontal Pole Left |
| 981 | Cerebelum 6 Right |
| 967 | Cerebelum 6 Left |
| 927 | Brain stem |
| 873 | Central Opercular Cortex Left |
| 826 | Central Opercular Cortex Right |
| 804 | Putamen |
| 788 | Superior Parietal Lobule Right |
| 767 | Precuneous Cortex |
| 697 | Supramarginal Gyrus, posterior division Right |
| 662 | Cerebelum Crus2 Left |
| 660 | Putamen |
| 648 | Hippocampus |
| 642 | Middle Temporal Gyrus, temporooccipital part Left |
| 635 | Cerebelum 4 5 Left |
| 616 | Temporal Occipital Fusiform Cortex Left |
| 610 | Middle Temporal Gyrus, temporooccipital part Right |
| 597 | Supramarginal Gyrus, anterior division Left |
| 592 | Hippocampus |
| 574 | Occipital Fusiform Gyrus Left |
| 565 | Planum Temporale Left |
| 561 | Cerebelum Crus1 Right |
| 553 | Cerebelum 4 5 Right |
| 531 | Parietal Operculum Cortex Right |
| 526 | Temporal Fusiform Cortex, posterior division Left |
| 518 | Parietal Operculum Cortex Left |
| 517 | Supramarginal Gyrus, anterior division Right |
| 506 | Inferior Frontal Gyrus, pars opercularis Left |
| 506 | Parahippocampal Gyrus, anterior division Right |
| 505 | Inferior Frontal Gyrus, pars triangularis Right |
| 505 | Supramarginal Gyrus, posterior division Left |
| 498 | Subcallosal Cortex |
| 480 | Middle Temporal Gyrus, posterior division Right |
| 440 | Planum Temporale Right |
| 428 | Paracingulate Gyrus Left |
| 427 | Inferior Frontal Gyrus, pars triangularis Left |
| 420 | Paracingulate Gyrus Right |
| 412 | Inferior Frontal Gyrus, pars opercularis Right |
| 407 | Temporal Occipital Fusiform Cortex Right |
| 394 | Vermis |
| 382 | Inferior Temporal Gyrus, temporooccipital part Left |
| 379 | Parahippocampal Gyrus, posterior division Left |
| 377 | Planum Polare Right |
| 363 | Superior Temporal Gyrus, posterior division Right |
| 361 | Temporal Fusiform Cortex, posterior division Right |
| 360 | Middle Temporal Gyrus, posterior division Left |
| 358 | Planum Polare Left |
| 354 | Superior Temporal Gyrus, posterior division Left |
| 353 | Parahippocampal Gyrus, anterior division Left |
| 342 | Amygdala |
| 337 | Superior Parietal Lobule Left |
| 327 | Amygdala |
| 319 | Parahippocampal Gyrus, posterior division Right |
| 318 | Frontal Operculum Cortex Left |
| 316 | Lateral Occipital Cortex, superior division Left |
| 309 | Heschl's Gyrus Left |
| 307 | Middle Temporal Gyrus, anterior division Left |
| 307 | Thalamus |
| 303 | Occipital Pole Left |
| 301 | Frontal Operculum Cortex Right |
| 299 | Cerebelum 8 Right |
| 292 | Middle Temporal Gyrus, anterior division Right |
| 282 | Heschl's Gyrus Right |
| 280 | Superior Temporal Gyrus, anterior division Left |
| 278 | Superior Temporal Gyrus, anterior division Right |
| 251 | Pallidum |
| 245 | Angular Gyrus Left |
| 244 | Caudate |
| 239 | Vermis |
| 226 | Temporal Fusiform Cortex, anterior division Right |
| 216 | Occipital Pole Right |
| 190 | Intracalcarine Cortex Right |
| 182 | Angular Gyrus Right |
| 178 | Frontal Medial Cortex |
| 176 | Caudate |
| 174 | Temporal Fusiform Cortex, anterior division Left |
| 173 | Middle Frontal Gyrus Left |
| 165 | Intracalcarine Cortex Left |
| 156 | Superior Frontal Gyrus Right |
| 146 | Cerebelum 3 Right |
| 144 | Occipital Fusiform Gyrus Right |
| 123 | Cuneal Cortex Right |
| 114 | Lateral Occipital Cortex, superior division Right |
| 106 | Cerebelum 7b Left |
| 104 | Middle Frontal Gyrus Right |
| 100 | Inferior Temporal Gyrus, anterior division Right |
| 91 | Inferior Temporal Gyrus, anterior division Left |
| 91 | Superior Frontal Gyrus Left |
| 85 | Cerebelum 9 Right |
| 80 | Inferior Temporal Gyrus, temporooccipital part Right |
| 78 | Cerebelum 10 Right |
| 74 | Accumbens |
| 73 | Accumbens |
| 72 | Cuneal Cortex Left |
| 71 | Vermis |
| 71 | Vermis |
